# Orthorexia Nervosa and Healthy Orthorexia in a Physically Active North American Population

**DOI:** 10.3390/nu16081107

**Published:** 2024-04-10

**Authors:** Jennifer L. Brodock, Helene Hopfer, Travis D. Masterson, John E. Hayes

**Affiliations:** 1 Sensory Evaluation Center, The Pennsylvania State University, University Park, Centre County, PA 16802, USAhxh83@psu.edu (H.H.); 2 Department of Food Science, The Pennsylvania State University, University Park, Centre County, PA 16802, USA; 3 Department of Nutritional Sciences, The Pennsylvania State University, University Park, Centre County, PA 16802, USA; travis.d.masterson@psu.edu

**Keywords:** physical activity, eating disorders, obesity, eating behavior

## Abstract

The Teruel Orthorexia Scale (TOS) defines two related but distinct constructs: Orthorexia Nervosa (OrNe), a pathological fixation on a healthy diet, and Healthy Orthorexia (HeOr), an interest in a healthy diet independent of psychopathology. Here, we (a) assessed both types of Orthorexia in a large North American sample using the TOS and (b) explored if engaging in regular physical activity was associated with a greater risk of Orthorexia. A cohort of physically active adults (*n* = 927; 41% men) completed the TOS, as well as the Rapid Assessment of Physical Activity (RAPA), to broadly assess aerobic physical activity level and participation in strength and/or flexibility training. As expected, scores for HeOr and OrNe differed between participants, with lower scores for Orthorexia Nervosa in our physically active non-clinical sample. Higher HeOr scores were associated with lower BMI, and this was true for both men and women. We also found that measures of Orthorexia were associated with self-reported physical activity: active adults reporting more aerobic physical activity had higher HeOr scores, with the most active men having the highest scores. Notably, adults who reported regular strength training had higher scores for both HeOr and OrNe, with men who strength trained showing higher OrNe scores than women. Here, those who participate in regular strength training are more likely to exhibit orthorexic behaviors, and this effect was more pronounced for men than women. Prior work has validated the TOS in young, primarily female samples of non-English speakers outside the United States: present data from an age-diverse, physically active, gender balanced sample support the use of TOS for measurement of Orthorexia Nervosa and Healthy Orthorexia in English speakers and suggest that more work is needed to assess potential gender differences in these constructs.

## 1. Introduction

Orthorexia was originally conceptualized by American physician Steven Bratman in 1997 as a pathological obsession with eating clean, healthy, or pure foods, but most subsequent work has occurred outside the United States. At present, Orthorexia is not included as a formal diagnosis in the most current version of the Diagnostic and Statistical Manual of Mental Disorders (DSM-5) due to an absence of sufficient evidence when the DSM was last updated in 2013. Still, clinical observations and a growing body of evidence suggest Orthorexia may be distinct from other eating disorders like ARFID (Avoidant/Restrictive Food Intake Disorder), BED (binge eating disorder), BN (Bulimia Nervosa), or AN (Anorexia Nervosa) [1]. As an eating pattern, Orthorexia is distinct from restrained eating and emotional eating [2]. Critically, unlike other eating disorders that often focus on the *amount* of food eaten, individuals with orthorexic tendencies tend to focus on the *quality* of food [3]; that is, they avoid foods that they feel are *unhealthy* or *impure* and spend a large amount of time thinking about procuring food. Such tendencies can lead to malnutrition and can impact personal relationships with others [4], resulting in impaired daily function and/or psychosocial harm. 

The most used measure of Orthorexia is the ORTO-15 questionnaire [5], including numerous modified or translated versions of it. As first described, ORTO-15 consists of 15 items that assess beliefs, attitudes, and habits about healthy eating using a 4-point scale; scores below 40 are thought to be indicative of Orthorexia while higher scores reflect more normal eating behavior. However, ORTO-15 has been widely criticized for psychometric flaws, including a lack of content validity as well as inconsistency in internal reliability [6,7,8]. Similarly, modified versions of the ORTO-15 have also proven unreliable [9,10]. Absent a psychometrically reliable measure or specific diagnostic code (i.e., DSM or ICD), it is hard to determine the prevalence of Orthorexia in the general population or to make comparisons across groups, such as investigating differences between men and women. 

Given the need for a more psychometrically valid tool to measure Orthorexia as a construct, the Teruel Orthorexia Scale (TOS) was recently developed using a large cohort of Spanish university students [11]. Based on exploratory factor analysis, the TOS separates Orthorexia into two related but distinct dimensions: Orthorexia Nervosa (OrNe) and Healthy Orthorexia (HeOr). In this conceptualization, OrNe suggests a problematic preoccupation with healthy eating while HeOr represents an interest in healthy eating that is independent of psychopathology (although some clinicians object to terming a nominally healthy behavior with “-orexia”, given the implied pathology with this suffix). The final TOS scale has 17 items: nine that assess HeOr (e.g., ‘I mainly eat foods that I consider to be healthy’) and eight that assess OrNe (e.g., ‘I feel guilty when I eat food that I do not consider healthy’). On the original TOS, agreement with individual prompts are made on a 4-point scale ranging from completely disagree to completely agree. HeOr and OrNe are calculated separately as the sum of the prompts, with higher scores indicating a higher degree of both HeOr and OrNe. The scale was initially validated in a Spanish population of university students and was found to be highly reliable, with a Cronbach’s alpha of 0.85 for HeOr and 0.81 for OrNe [11]. Recent translations of the TOS have also been used to assess Orthorexia in German and Lebanese populations, with reliability for both subscales ranging between 0.83–0.88 [12].

Use of the TOS in a large, diverse North American sample is currently lacking from the published literature, and Depa and colleagues specifically called for further validation in non-Spanish and non-university populations—we help address this gap here. Specifically, as part of a larger study on consumer behavior published previously, we used the TOS to assess HeOr and OrNe in a physically active North American population. In this secondary analysis of existing data, we explore if Orthorexia is correlated with increasing levels of physical activity or self-reported adiposity. We also test the effect of gender on HeOr and OrNe scores, as previous studies on Orthorexia using the TOS [2,13] and other instruments [14] have conflicting reports on the effects of gender. 

## 2. Materials and Methods

### 2.1. Overview

Recently, we reported on consumer attitudes and preferences for dark chocolate milk that varied in added sugar, fat level, and sweetener type using a standard market research technique called choice-based conjoint analysis [15]. Critically, our adult participants in that study also completed the Teruel Orthorexia Scale (TOS) and the Rapid Assessment of Physical Activity (RAPA) and self-reported height and weight, exercise type and duration, importance of protein in their diet, and some brief demographics in that study. Given the absence of TOS data in an age-diverse North American sample in the published literature, we leveraged existing data here for secondary analysis, focusing here on TOS, RAPA, BMI, and demographics. These data are publicly available via the link provided at the end of this paper.

### 2.2. Participant Recruitment and Characteristics

**Figure 1:**
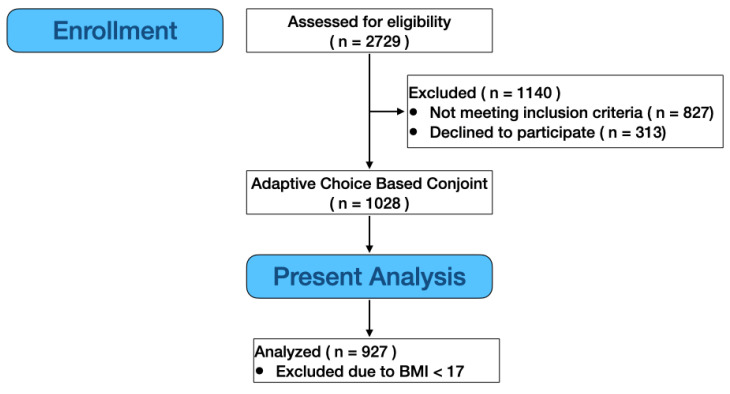
Flow chart for assessing participant eligibility and inclusion in analysis. Data from the main Adaptive Choice-Based Conjoint (ACBC) study are reported in [15]; here, we used data from 380 men and 547 women after excluding those with BMIs under 17 (see text).

Data from 927 adult participants (41% men and 59% women aged 18–45) are reported here. They were recruited from the mid-Atlantic region of the United States (NY, NJ, MD, D.C., PA, and VA) by a third-party market research agency (Dynata LLC, Shelton, CT, USA) to participate in our adaptive choice-based conjoint analysis study of various chocolate milk formulations; they qualified for the study if they were (a) aged 18 to 45 years old and (b) exercised at least 3 times per week (labeled as ACBC in Figure 1). Informed consent was documented on the first screen of the survey via yes/no questions, and participants were compensated for their time according to the standard scheme used by Dynata on all projects. For the present analyses, we cleaned the data with an additional step: participants were excluded from analysis if their BMI, calculated from self-reported heights and weights, was less than 17. We selected this cutoff for adults as it may (a) arise from data entry error when reporting their height and weight (e.g., entering kg instead of lbs) or (b) be indicative of another eating disorder. However, as a further check, we also performed sensitivity analysis: when BMIs that are less than 17 were included, the same overall patterns of results were seen. All procedures, including screening and recruitment, were deemed exempt from full Institutional Review Board (IRB) review by professional staff in the Office of Research Protections (ORP) at The Pennsylvania State University (STUDY00012575).

### 2.3. Instruments and Data Collection

Data from 927 adults are reported here. All data were collected using specialized software [16] for collection and analysis of adaptive conjoint analysis (Lighthouse Studio from Sawtooth Software (Academic Research, version 9.8.1, Provo, UT, USA)). At the end of the product-focused conjoint study, participants completed two validated questionnaires on physical activity and Orthorexia, along with brief demographics. These data are the focus of the current manuscript.

The RAPA is a two-part tool [17] to assess the intensity and duration of physical activity (RAPA1) and participation in strength and flexibility activities (RAPA2). RAPA1 is scored from 1–7, where a score of 5 or below is considered under-active. RAPA2 is scored from 0–3, where zero indicates no strength or flexibility activity, a score of 1 corresponds to just strength activities, 2 to only flexibility activities, and a score of 3 equates to both strength and flexibility activities at least once a week.

The TOS is used to assess healthy and unhealthy orthorexic behaviors. As described above, the TOS measures both Healthy Orthorexia (HeOr) and Orthorexia Nervosa (OrNe) via 17 statements participants endorse. Here, we modified the original four-point category scale (0—completely disagree to 3—completely agree) described in [11] to a five-point Likert scale (0—completely disagree to 4—completely disagree) by adding a neutral option for participants. This was done to better align with best practices for the use of Likert-type scales (see [18,19]), but this modification also means that in our study, HeOr was scored from 0–36 and OrNe from 0–32, so raw means should not be directly compared to prior reports.

### 2.4. Data Analysis

All analyses were performed using RStudio (version 1.2.5033) and R (version 3.6.2). Scores were calculated for the RAPA and TOS following published guidelines, save Likert scale modifications noted previously. Body mass index (BMI) was calculated using self-reported height and weight. Non-parametric (Spearman) correlations were calculated using the ggpairs function for age, BMI, HeOr, OrNe, RAPA1, and RAPA2 [20] separately for women (coded as 0) and men (coded as 1). Regression models with interaction terms were performed to formally test for gender differences observed in the variable pairs with significant correlations. A two-way ANOVA was performed with RAPA2 scores by HeOr and gender and with the RAPA2 scores by OrNe and gender. Although typically coded as values from 0 to 3, RAPA2 scores are not strictly ordinal measures, so they were included in the ANOVA model as a factor with 3 levels: no activity (score of 0), lifting or stretching but not both (score of either 1 or 2), and lifting and stretching (score of 3). Post-hoc means comparison with Tukey’s HSD (*p* < 0.05) with the agricolae package [21] was used to determine the levels of RAPA2 by HeOr and OrNe that significantly differed from each other. Cronbach’s alpha was calculated for RAPA1, HeOr, and OrNe using the psych package [22] to measure the internal consistency of the scales. Other packages included tidyr [23] and ggplot2 [24]. Statistical significance was defined as *p* < 0.05.

## 3. Results

### 3.1. Demographics and Questionnaire Scores

The mean age for our sample is 31.6 years (±7.9 SD). BMI was calculated from self-reported height and weight; the mean BMI of the sample described here is 25.6 ± 6.5. The mean HeOr and OrNe scores for this sample are 23.0 ± 7.0 and 14.1 ± 7.2, respectively. The mean RAPA1 and RAPA2 scores for physical activity are 6.1 ± 1.4 and 1.9 ± 1.2, respectively. See Table 1 for ranges and gender-specific means. 

### 3.2. Spearman Correlations

**Figure 2:**
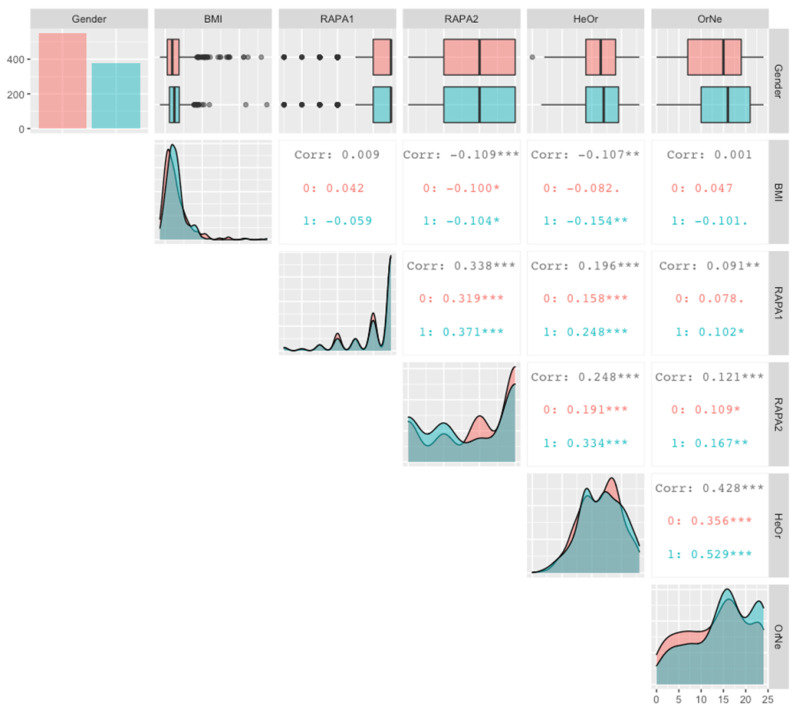
Scatter matrix showing Spearman’s correlations calculated across the entire cohort (in black), and separately by gender. Women were coded as 0 and are shown in salmon, men were coded as 1 and are shown in teal. For each variable, the top row shows a summary of descriptive statistics as box-and-whisker plots, and the diagonal shows the distribution for each variable, separately by gender. Within individual squares, the Spearman correlations are summarized for each pair of variables across the entire cohort (in black) and separately for women (salmon; 0) and men (teal; 1). Within a square, significant correlations are denoted with stars, where ***, **, and * indicate *p* values of <0.001, <0.01, and <0.05, respectively. For example, HeOr was negatively correlated with BMI, but this effect was only significant in men, while HeOr was positively correlated with RAPA2 in both women and men. See text for additional information on pairwise relationships.

As shown in Figure 2, there are significant correlations between 8 of the 10 pairs of variables; Spearman correlations were used instead of Pearson correlations as it is less sensitive to outliers and thus better suited for an initial, exploratory analysis of associations. RAPA2 and HeOr each show a significant negative correlation with BMI. However, when separated by gender, the simple Spearman correlation between HeOr and BMI was only significant for men. Across the entire sample, RAPA2, HeOr, and OrNe are each positively correlated with RAPA1. However, when calculated separately by gender, the correlation between OrNe and RAPA1 only remained significant for men; the gender-specific correlations for RAPA2-RAPA1 and RAPA2-HeOr remained significant when separated by gender. Similar to RAPA1, the correlations between RAPA2 and both HeOr and OrNe are significant across the entire sample. Again, the correlations are stronger in men than in women, although significant for both. Finally, OrNe and HeOr are positively correlated with each other, as expected, and the correlation appears to be much stronger in men than in women.

### 3.3. Regression Models to Formally Test Tentative Gender Interactions Seen in Correlation Matrix

Regression models with interactions were run for the 8 significant correlations observed to explore if physical activity and gender could predict both types of Orthorexia scores and BMI. Only two models had significant gender interactions; thus, multiple regression was used to test if physical activity and gender could predict HeOr scores. 

**Figure 3:**
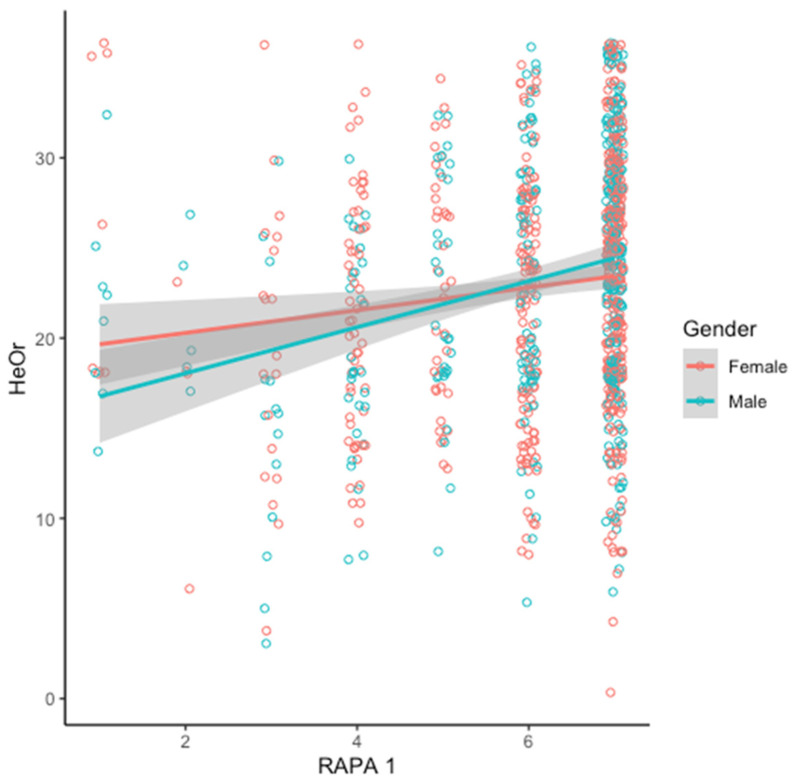
Healthy Orthorexia score (HeOr) increases with the intensity and duration of aerobic physical activity (RAPA1) for men (teal) and women (salmon), but the relationship is stronger in men (see text for details of model).

As visualized in Figure 3, the relationship between RAPA1 and HeOr varies by gender. The overall model is significant (R^2^ = 0.04, F(3923) = 12, *p* < 1.037 × 10^−7^), and RAPA1 scores significantly predicts HeOr (β = 0.63, *p* = 0.004). There is no main effect of gender (β = −3.54; *p* = 0.09), but the interaction between RAPA1 and gender is found to be significant (β = 0.65, *p* = 0.0489). Collectively, this indicates that HeOr scores increase with the intensity and duration of aerobic activity for both women and men, but the slope is steeper for men.

**Figure 4:**
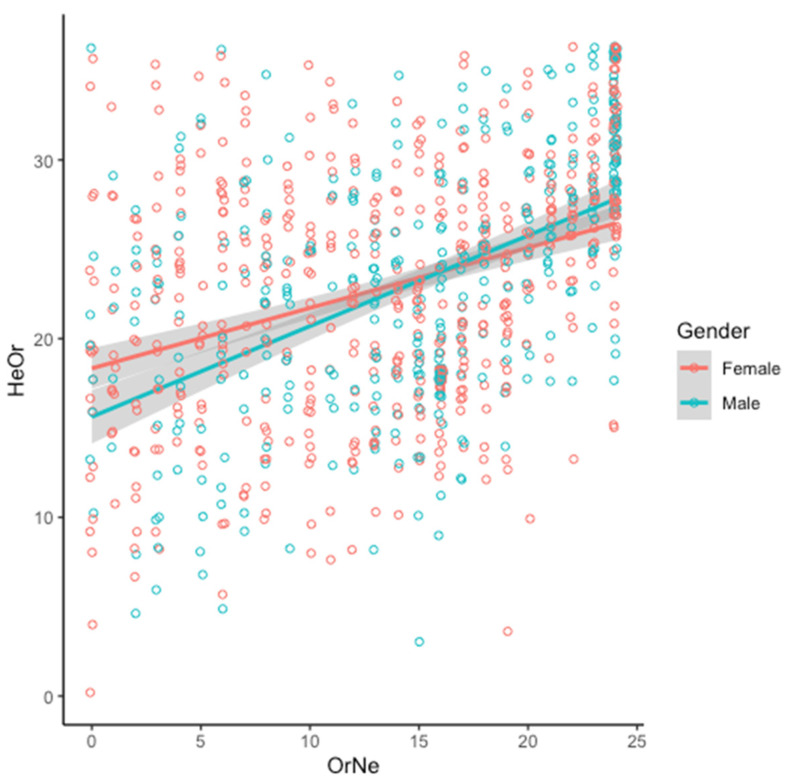
Healthy Orthorexia score (HeOr) increases with Orthorexia Nervosa score (OrNe) for both men (teal) and women (salmon), but the relationship is stronger in men (see text for details of model).

As visualized in Figure 4, the relationship between OrNe and HeOr varies slightly by gender. The overall model is significant (R^2^ = 0.18, F(3923 = 66.09, *p* < 2.2 × 10^−16^), and gender (β = −2.71, *p* = 0.005 and OrNe (β = 0.34, *p* < 2 × 10^−16^) are both significant predictors of HeOr (β = 19.03, *p* < 2 × 10^−16^). Also, the interaction between OrNe and gender is significant (β = 0.17, *p* = 0.005).

### 3.4. Two-Way ANOVA

Despite being coded as values from 0 to 3 in the original RAPA2 scoring instructions, thoughtful consideration reveals that the scores on the scale are not strictly ordinal: that is, lifting only and stretching only. Values of 1 versus 2 are not truly hierarchical. Accordingly, for the analysis here, the original 4-point scale was collapsed into 3 levels. These categories were no activity (=0), lifting or stretching (but not both) (=1 or 2), and lifting and stretching (i.e., both activities) (=3). 

**Figure 5:**
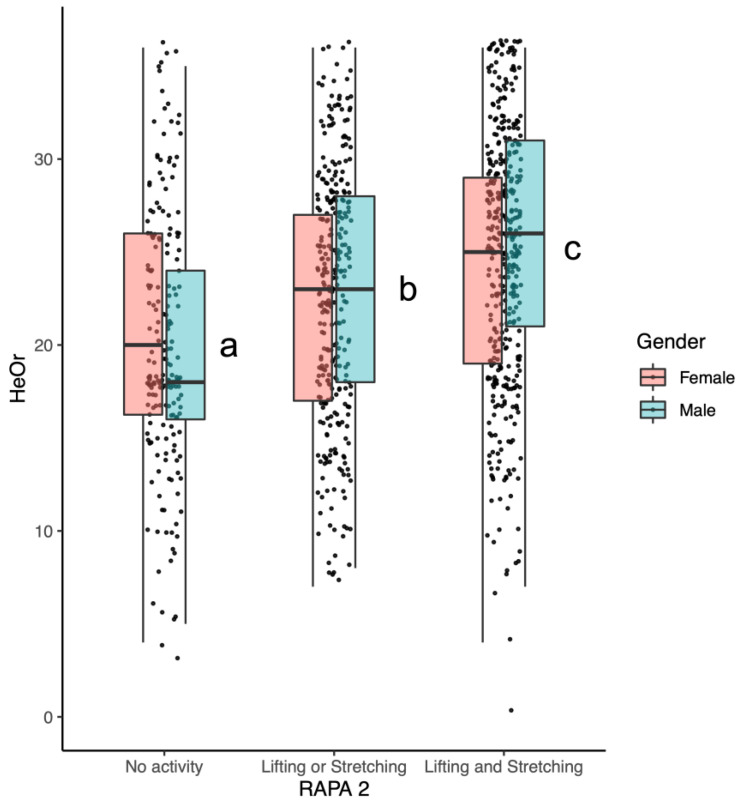
Mean Healthy Orthorexia (HeOr) scores differed by RAPA2 activity categories, but this relationship was not affected by gender. Activities with different letters are significantly different.

As shown in Figure 5, significant differences in HeOr scores by activity type were observed (F(2921) = 29.6, *p* < 3.49 × 10^−13^); conversely, the main effect of gender and the gender by activity type interaction is not significant. A comparison via Tukey’s HSD shows that mean Healthy Orthorexia scores (HeOr) differ with activity type: lifting *and* stretching (=24.7) is greater than lifting *or* stretching (=22.7), which in turn is greater than no activity (=20.2).

**Figure 6:**
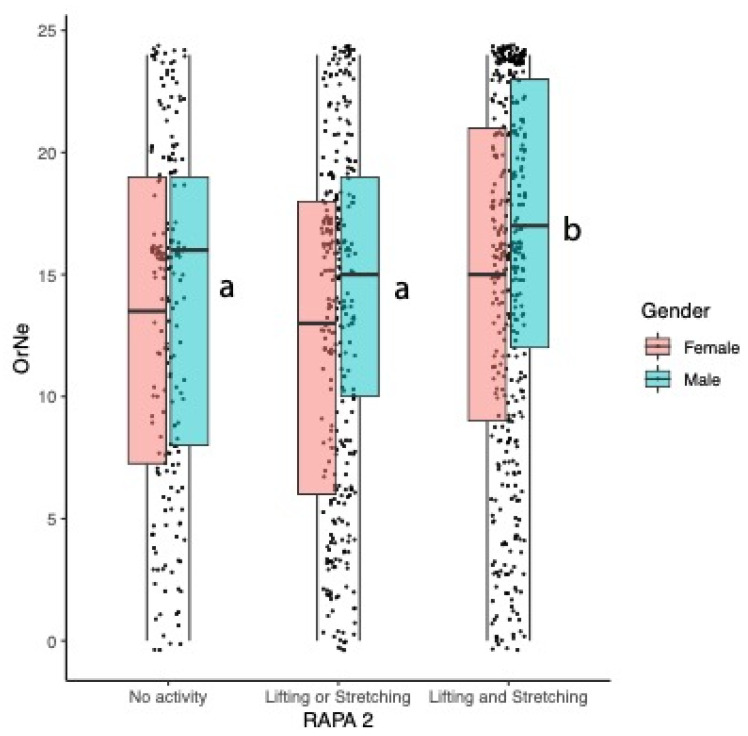
Mean Orthorexia Nervosa (OrNe) scores differed by RAPA2 activity categories: OrNe scores were higher in those engaged in lifting and stretching. Activities with different letters indicate significant differences between activity levels. Within an activity type, means for men were always higher than means for women, but the activity type by gender interaction was not significant.

As shown in Figure 6, significant differences are also observed for OrNe scores by activity type (F(2921) = 8.9, *p* = 0.00015), and the main effect of gender is significant (F(1921) = 13.8, *p* = 0.0002), with men consistently having greater scores than women; the gender by activity type interaction is not significant. A comparison via Tukey’s HSD shows mean OrNe scores differ by activity type: lifting and stretching = 15.2 is greater than lifting or stretching = 13.1 and no activity = 13.4, which are not different from each other. 

### 3.5. Internal Reliability

Internal reliability was calculated using Cronbach’s alpha for both TOS components: HeOr and OrNe. The Healthy Orthorexia subscale consisted of 9 items and was found to be highly reliable (α = 0.86), as was the 8 item Orthorexia Nervosa subscale (α = 0.90). 

### 3.6. Predicting BMI from HeOr and OrNe Scores

Here, BMI was calculated from the participant’s self-reported height and weight. As reported above, Spearman’s correlations showed a significant negative correlation between BMI and Healthy Orthorexia (r = −0.11) and no correlation between BMI and Orthorexia Nervosa (r = 0.001) when calculated across the entire cohort of men and women. However, this simple bivariate approach does not account for the observed positive relationship between Healthy Orthorexia and Orthorexia Nervosa. 

In multiple regression testing the effect of both subscales on BMI simultaneously, the overall model is significant (R^2^ = 0.017, F(2924) = 7.94, *p* = 0.0004), and both subscales are significant predictors: HeOr (β = −0.13, *p* = 0.0002) is a negative predictor of BMI while OrNe (β = +0.09, *p* = 0.006) is a positive predictor. That is, looking across both women and men, a 10-point increase in Healthy Orthorexia score predicted a 1.3-point decrease in BMI, while a 10-point increase in Orthorexia Nervosa predicted a 0.91-point increase in BMI. Leveraging our large sample size, we further explored if there were gender-specific relationships with BMI by running models separately for men and women. When a multiple regression model with both subscales of the TOS was run in only women, both HeOr (β = −0.12, *p* < 0.01) and OrNe (β = +0.11, *p* < 0.05) were significant predictors. When a parallel model in just men was run, OrNe was not associated with BMI (β = +0.05, *p* = 0.27), but HeOr remained a significant negative predictor (β = −0.12, *p* < 0.01).

## 4. Discussion

The possible ranges for Orthorexia Nervosa (OrNe) and Healthy Orthorexia (HeOr) in our study were 0–32 and 0–36, respectively. As expected with a non-clinical sample, the mean for Healthy Orthorexia was greater than the mean for Orthorexia Nervosa, consistent with prior work. Previously, Barrada and Roncero reported mean scores in their initial paper of 12.52 for HeOr and 3.57 for OrNe [11], while means for our sample were 23.0 for HeOr and 14.1 for OrNe. However, caution is warranted in direct comparisons with previous reports; that is, we observed higher mean scores than Barrada and colleagues in part because we included a true midpoint on our Likert scale, consistent with recommended practices [18,19]. Still, if mean scores are re-expressed as a simple fraction of the total possible range in each study, our data show higher values for both Healthy Orthorexia (0.64 versus 0.46) and Orthorexia Nervosa (0.44 versus 0.15) than the study of Barrada and colleagues. These differences may be due to multiple factors, including culture (Spain versus mid-Atlantic USA), age differences between the samples (mean 24.0 versus 31.6 years), gender split (24% men versus 41% men), or study inclusion criteria (university students versus physically active adults). We believe this last factor may be especially salient given the relationships observed here between physical activity and Orthorexia, and our recruitment of a physically active cohort. In 2020, Strahler and colleagues [12] compared published Orthorexia scores using the TOS in German and Lebanese cohorts. They found that there was a higher risk of Orthorexia Nervosa in the Lebanese sample than in the German sample, which they attributed to sociocultural differences in their environments [12]. The reported means for their study, again re-expressed as fractions of the possible range to account for the addition of a midpoint, were also much lower than values seen here for both Healthy Orthorexia (0.39 and 0.43 versus 0.64 here) and Orthorexia Nervosa (0.33 and 0.14 versus 0.44 here). Thus, we can speculate that these consistent differences are probably most attributable to the physical activity recruitment criterion for our study but also possibly gender, as our study had more men than prior samples, although we cannot rule out cultural and age differences. Accordingly, while present data make a novel contribution by documenting evidence of Orthorexia in a North American sample, we also caution that present data should not be used as normative values for the United States, given that our recruitment criteria enriched our sample with physically active individuals.

Previously, Depa et al. [13], Barrada and Roncero [11], and Barthels et al. [2] all reported a weak but significant negative correlation between BMI and HeOr scores (r = −0.15, −0.06, −0.14). Conversely, OrNe showed a weak positive correlation with BMI in only one of the three previous studies (r = +0.11; Depa et al. [13]). Here, we observed a similar pattern: a weak but significant negative correlation between BMI and Healthy Orthorexia and a weak positive correlation between BMI and Orthorexia Nervosa. When split by gender, we found simple bivariate correlations between Healthy Orthorexia and Orthorexia Nervosa and BMI were both stronger in men. Furthermore, when both Healthy Orthorexia and Orthorexia Nervosa were included simultaneously in a multiple regression model, they were both significant predictors of BMI, with Healthy Orthorexia as a negative predictor and Orthorexia Nervosa as a positive predictor. These findings are consistent with Depa and colleagues, who also found Healthy Orthorexia to be a significant negative predictor of BMI. However, when this model was run separately for men and women, only HeOr scores remained a significant predictor of BMI for men; both HeOr and OrNe scores remained significant predictors of BMI for women. Depa and colleagues [13] did not report any effects of gender in their findings, but their sample was limited to only 18% men, and therefore, it seems unlikely they were powered to effectively test for gender effects. Finally, we should note that self-reported heights and weights were used in our study to calculate BMI values, so we cannot rule out reporting bias; further, if any such bias is present in our sample, it may not be consistently distributed across the sample if reporting bias varies with adiposity.

The TOS is a recently introduced tool to measure Orthorexia in non-clinical populations, given the widely recognized flaws in earlier measures. The TOS has been compared to other measures of eating behavior, including food choice motives, restrained eating, and emotional eating, as well as affective responses [2,13]; however, it has not been associated with physical activity previously. Here, we find that both self-reported intensity and duration of aerobic activity and self-reported strength and flexibility training were significantly positively correlated with both the Healthy Orthorexia and Orthorexia Nervosa subscales of the TOS. Notably, however, there was a stronger correlation between HeOr scores and aerobic activity than OrNe scores and aerobic activity in our sample. Further, this pattern was more pronounced in men (see Figure 3), suggesting that as men become more physically active, they may also become more preoccupied with healthy eating, at least relative to women who are equally physically active. Conversely, underactive women tended to have higher Healthy Orthorexia scores relative to underactive men, which may speak to gendered food norms and cultural expectations independent of physical activity. This is largely consistent with recent work from Strahler and colleagues, who reported a significant sex effect for the Healthy Orthorexia subscale of the TOS [12].

Here, we also found that self-reported strength and flexibility training predicted differences in HeOr scores, with those who reported engaging in both strength and flexibility training having the highest HeOr scores. Self-reported training also predicted OrNe scores, with those self-reporting strength training and flexibility training again having the highest scores. As highlighted in the introduction, the idea of ‘clean eating’ refers to the *quality* of one’s diet rather than the *amount* of food or calories consumed. The relationship we observed between an increase in aerobic activities/strength training and Orthorexia Nervosa suggests that as people choose to become more active, they may also become more focused on diet quality. This interpretation is consistent with other work from our team, where we found that consumers who participate in strength training regularly also reported being more concerned with protein intake and the amount of calories they consume in their diet. Further, although there was not a significant main effect of gender for self-reported strength and flexibility training and Healthy Orthorexia (Figure 5), the plot also shows a clear jump in HeOr when men partake in lifting or stretching, relative to no activity. This suggests that men who are inactive may have less focus on healthy eating habits than women who are equally inactive, but this disregard among men disappears if they choose to become active, an interpretation that is also conceptually consistent with the gendered interaction seen in the aerobic activity data (Figure 3). This is similar to results from Strahler and colleagues, who found HeOr to be higher in men in both the German and Lebanese samples but found no differences between genders in regards to OrNe [12]. In the initial work with the TOS in Spain [2,11,13], no differences in gender were reported, but presumably, this may be due to the low number of men participating in their initial studies (24%, 18.5%, 17.9%). In contrast, our sample was both larger and more gender-balanced, with 41% men. 

Here, Orthorexia Nervosa significantly differed between men and women, with men having higher OrNe scores. Self-reported strength and flexibility training was a significant predictor of OrNe, suggesting that those who participate in strength training are more likely to focus on diet quality. This is not entirely surprising as regular strength trainers often carefully track their macronutrients, especially protein, in order to aid in their physique goals. For example, our group previously found that 46% of consumers who regularly participated in strength training also reported engaging in cutting and bulking cycles [15]. These cycles refer to periodic patterns of intake some athletes engage in, where they eat in a caloric surplus for a period of time to gain muscle mass (bulking) and then slowly cut calories down to a deficit to lose fat (cutting). Engaging in bulking and cutting cycles usually requires an extreme degree of attention to diet in order to ensure adequate calories are consumed for either cycle. Thus, it stands to reason that those engaging in these eating cycles would have higher healthy orthorexic tendencies when given the TOS. 

## 5. Conclusions

In a roughly gender-balanced sample of physically active adults in North America, we find that Healthy Orthorexia and Orthorexia Nervosa are correlated with each other, and the effect is stronger in men. Whether these relationships hold in less physically active individuals remains unknown. Further, we also find evidence that the two subscales of the TOS measure different constructs of eating behavior, as they have opposing relationships with BMI in this sample: specifically, Healthy Orthorexia is negatively correlated with BMI, while Orthorexia Nervosa is positively correlated with BMI. Finally, we find preliminary evidence that both subscales are associated with self-reported duration of physical activity as well as self-reported strength and flexibility training, although additional work would be needed to confirm this relationship in a less active general population sample.

## Figures and Tables

**Table 1:** Participant mean and observed range of demographics and questionnaire scores by gender.

Gender	Age	BMI	HeOr	OrNe	RAPA1	RAPA2
Male	32.7	25.7	23.3	15.1	6.1	1.7
Female	30.8	25.6	22.9	13.4	6.1	1.9
Range	18–45	17.1–74.9	0–36	0–32	1–7	0–3

## Data Availability

The anonymized data used for this study are available for download at https://scholarsphere.psu.edu/resources/659e2a54-d4cb-4391-bc61-b3655ab13042 (accessed on 15 April 2021).
